# Drought Stress Inhibits the Accumulation of Rotenoids and the Biosynthesis of Drought-Responsive Phytohormones in *Mirabilis himalaica* (Edgew.) Heim Calli

**DOI:** 10.3390/genes15121644

**Published:** 2024-12-21

**Authors:** Shiyi Zhang, Jiaqi Gao, Xiaozhong Lan, Linfan Zhang, Weipeng Lian, Chenglin Wang, Zhanyun Shen, Xiang Li, Juan Liu

**Affiliations:** 1 State Key Laboratory for Quality Ensurance and Sustainable Use of Dao-di Herbs, National Resource Center for Chinese Materia Medica, China Academy of Chinese Medical Sciences, Beijing 100700, Chinalinfanz@126.com (L.Z.); m18222616303@163.com (X.L.); 2 School of Pharmaceutical Sciences, Zhejiang Chinese Medical University, Hangzhou 310053, China; 3 Medicinal Plants Research Centre, Xizang Agricultural and Animal Husbandry University, Nyingchi 860000, China; 4 College of Traditional Chinese Medicine, Zhejiang Pharmaceutical University, Ningbo 315500, China

**Keywords:** *Mirabilis himalaica*, drought stress, rotenoids, abscisic acid, auxin, jasmonate, transcriptome

## Abstract

**Background:** *Mirabilis himalaica*, distributed in the high-altitude, arid, and semi-arid regions of Xizang, exhibits great tolerance to drought, which is rich in rotenoids and other secondary metabolites. It is still unknown, though, how drought stress influences rotenoid synthesis in *M. himalaica*. **Methods**: In this study, the calli of *M. himalaica* were subjected to 5% PEG6000 for 0, 20, and 40 h and divided into control group (CK), mild-drought-treated group (M), and high-drought-treated group (H), respectively. We then analyzed the relative content of three main rotenoids in *M. himalaica* using high-performance liquid chromatography–electrospray ionization–tandem mass spectrometry (HPLC-ESI-MS/MS). **Results**: Our findings demonstrated that the content of rotenoids was significantly reduced under drought stress. Transcriptome analysis subsequently revealed 14,525 differentially expressed genes (DEGs) between the different treatments. Furthermore, these DEGs exhibited enrichment in pathways associated with isoflavone biosynthesis and hormone signaling pathways. Key genes with decreased expression patterns during drought stress were also found to be involved in rotenoid accumulation and drought-responsive phytohormone signaling, including abscisic acid (ABA), auxin (IAA), and jasmonic acid (JA). **Conclusions**: These findings elucidate the molecular processes of drought resistance in *M. himalaica* and shed light on the relationship between rotenoid production and drought stress in *M. himalaica*.

## 1. Introduction

*Mirabilis himalaica*, commonly known as *Oxybaphus himalaicus* Edgew, is a perennial herb from the Nyctaginaceae family. The root of *M. himalaica* is utilized as a traditional medicine known as “Bazhu”, which is primarily found in eastern Xizang and the Himalayas in China. The main active ingredients in *M. himalaica* are rotenoids, which can also act as important chemotaxonomic markers for the plant due to their high content [1]. Modern pharmacological evaluations of this herb’s rotenoids revealed that boeravinone E demonstrated spasmolytic activity [2], whereas boeravinone B exhibited anticancer activity by triggering internalization and destruction of inactivated EGFR and ErbB2 in human colon cancer cells [3]. Furthermore, a number of studies have revealed chalcone synthase (CHS) and chalcone isomerase (CHI) genes play an important role in rotenone biosynthesis [4,5], with CHS serving as a rate-limiting enzyme in this process [6]. Controlling the rotenoid production is crucial in *M. himalaica*, but it is not yet unclear how environmental factors affect the amount of rotenoid in *M. himalaica*.

Drought stress, as an essential abiotic stress factor, influences plant growth and development significantly. Plants cope with drought by regulating secondary metabolite pathways and hormone synthesis to maintain overall function [7]. For example, drought stress increased the total flavonoid content of three Achillea species [8]. Both rutin and quercetin levels in *Hypericum brasiliense* increased in response to drought stress [9]. It has also been discovered that a CCCH-type transcription factor, PuC3H35, has a role in the production of proanthocyanidins (PAs) and lignin in the root of *Populus ussuriensis* during drought stress [10]. Abscisic acid is a key signal for plants to respond to drought, causing a variety of physiological processes such as stomatal closure, root system modulation, soil microbial community organization, transcriptional and post-transcriptional gene expression activation, and metabolic changes [11]. Additionally, auxin could participate in the positive regulation of drought stress resistance, through Aux/IAA proteins, ABA-responsive gene expression, ROS metabolism, and metabolic homeostasis [12,13]. Jasmonic acid could contribute to drought stress signaling, such as the activation of some drought-responsive genes, the modulation of Ca^2+^ ions, and the promotion of some secondary plant metabolic pathways such as flavonoids and terpenoids, which are required for plant adaptation to stress conditions [14]. *M. himalaica*, which is mostly found in high-altitude desert and semi-arid regions of Xizang [15], has distinctive traits such as extreme drought, UV radiation, substantial diurnal temperature changes, and limited precipitation. Therefore, *M. himalaica* is able to tolerate drought and other stresses. Currently, research on *M. himalaica* focuses on its chemical composition and pharmacological properties. However, there have been few studies on the effect of drought stress on the control of *M. himalaica* flavonoid production, particularly its active component rotenoids.

In this study, *M. himalaica* calli were utilized as experimental materials, and drought conditions were simulated using PEG6000. The relative levels of three rotenoids were then examined in drought-treated *M. himalaica* calli using ultra-high-performance liquid chromatography–electrospray ionization–tandem mass spectrometry (UPLC-ESI-MS/MS). UPLC-ESI-MS/MS results showed that a 20 h treatment with 5% PEG6000 significantly reduced rotenoid production in *M. himalaica*. To better understand *M. himalaica*’s drought resistance mechanism, we looked into the potential phytohormone network connected with its response to drought stress. This study elucidates the probable molecular pathways of drought resistance in *M. himalaica* and provides a scientific foundation for cultivating and enhancing rotenoid output.

## 2. Materials and Methods

### 2.1. Plant Materials

**Figure 1:**
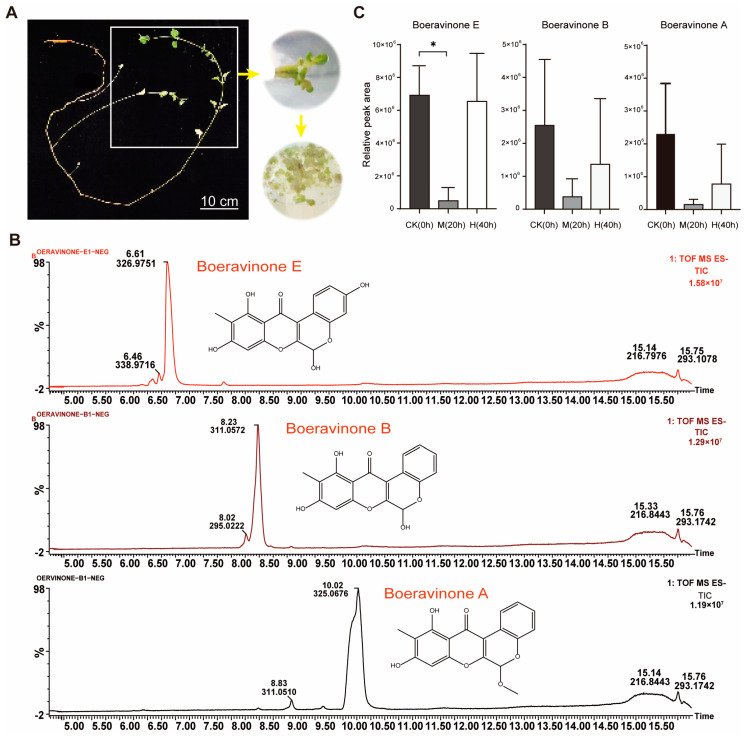
Analysis of the relative content of three rotenoids. (**A**) *M. himalaica* leaves before and after callus induction; (**B**) total ion current plots of three rotenoids; (**C**) the relative content of boeravionon E, boeravionon B, and boeravionon A. Statistical analysis was performed using GraphPad Prism (9.5.1) software. The significance of differences between groups was assessed using one-way ANOVA. Asterisks * indicate statistical significance at *p* < 0.05.

The leaves of *M. himalaica* were sterilized using 75% ethanol and 2% effective chlorine solutions for 30 s and 15 min, respectively; subsequently, they were cultured in Mu-rashige and Skoog (MS) medium containing 0.3 μg·L^−1^ of 2, 4-dichlorophenoxyacetic acid (2,4-D), 0.8 μg·L^−1^ of naphthalene-1-acetic acid (NAA), and 1.2 μg·L^−1^ of 6-Benzylaminopurine (6-BA) for callus induction (Figure 1A). The calli were incubated on MS medium supplemented with 5% PEG6000 and designated as the control group (CK, 0 h), mild drought treatment (M, 20 h), and high treatment (H, 40 h); each group consisted of three biological replicates. Immediately following the completion of the treatment, a portion of the sample was frozen in liquid nitrogen and subsequently stored at −80 °C for the purpose of RNA extraction. Following freeze-drying, the remaining portion of the sample was dissolved in an equal volume of pure methanol and extracted by ultrasonication for 30 min. The solution was then filtered through a 0.22 μm PTFE membrane in preparation for UPLC-ESI-MS/MS analysis.

### 2.2. Chromatography Mass Spectrometry Conditions

For the determination of the relative content of three rotenoids, an Absciex 6500QTRAP triple quadrupole mass spectrometer and a Waters ACQUITY UPLC BEH C18 column (2.1 × 100 mm, 1.7 μm) were used. The mobile phase consisted of 0.1% (*v*/*v*) formic acid in acetonitrile (A) and 0.1% formic acid in water (B), following this gradient: 95–50% A over 0–7 min, 50–30% A from 7 to 12 min, 30–5% A between 12 and 15 min, and 5–95% A from 15 to 16 min. The flow rate was maintained at 0.5 mL/min, the injection volume was 1 μL, and the column temperature was controlled at 40 °C.

Negative ion scan mode and MSE Continuum acquisition mode were employed using an ESI ionization mode. The capillary voltage was set at 2.0 KV/0.5 KV for positive and negative ions, respectively; cone voltage was maintained at 40 V. Nitrogen was used as the solvent removal gas at a flow rate of 900 L/h, and the temperature was set at 450 °C. The scan range extended from *m*/*z* 50 to 1500 Da, with argon as the collision gas. Trap collision energy settings were 6 eV during low-energy scans and ranged from 40 to 70 eV for both positive and negative ions during high-energy scans. Data acquisition and analysis were performed using MassLynx V4.2 software and UNIFI 1.9 software, respectively.

### 2.3. RNA Extraction

Total RNA was extracted from ultra-frozen calli using RNAiso Plus (TaKaRa, Kyoto, Japan). Samples were ground in liquid nitrogen, mixed with RNAiso Plus in a 50:1 ratio, and incubated at room temperature for 5 min. Following initial centrifugation, the supernatant was treated with chloroform, mixed, and centrifuged again to clarify. Isopropanol was added to the supernatant, which was then left to precipitate RNA before a final centrifugation. Ethanol was used to wash the RNA, which was then dried and dissolved in RNase-free water for analysis.

### 2.4. Transcriptome Sequencing

Following total RNA isolation, eukaryotic mRNA was enriched using Oligo(dT)18-coated magnetic beads. A fragmentation buffer was then added to cleave the mRNA into shorter fragments. The first cDNA strand synthesis employed random hexamers as primers. This was followed by the introduction of a reaction mixture comprising buffer, dNTPs, RNase H, and DNA polymerase I for second cDNA strand synthesis. The resultant double-stranded cDNA was purified with the QiaQuick PCR kit and eluted in EB buffer. Subsequent steps included end repair, poly(A) tailing, and sequencing linker ligation. Fragment size selection was conducted via agarose gel electrophoresis, followed by PCR amplification. The prepared sequencing library was then sequenced on an Illumina HiSeq X platform. The data have been deposited in the National Genomics Data Center (NGDC)’s Genome Sequence Archive, under accession number CRA016807, and are publicly accessible at https://bigd.big.ac.cn/gsa/browse/CRA016807, accessed on 3 June 2024.

### 2.5. Analysis of Endogenous ABA, IAA, and JA

Retention times of target compounds were obtained from the Human Metabolome Database (HMDB, https://hmdb.ca) by cross-referencing their molecular weights, as well as their MS/MS spectra (both precursor and fragment ions), under conditions similar to those in our experiments. These retention times were then compared with experimental data for identification. The peak areas corresponding to these retention times were automatically integrated using MassLynx V4.2 software. Relative quantification of the target compounds was performed by comparing the integrated peak areas across the CK, M, and H groups. The information of three endogenous plant hormones is shown in Table S1. Statistical analysis was performed using GraphPad Prism (9.5.1) software. The significance of differences between groups was assessed using one-way ANOVA. Asterisks * and ** indicate statistical significance at *p* < 0.05 and *p* < 0.01, respectively.

### 2.6. Bioinformatics Analysis of Transcriptome Data

Raw data from each sample were filtered using fastp (0.9.1) software based on several of the following criteria: fewer than 5 N bases, a 4 bp sliding window with an average quality below Q20, Q20 above 0 for all reads, automatic adapter removal while retaining non-adapter sequences, and a minimum length of 75 bp. Clean data were then aligned to the ribosome database to extract unmapped reads from each sample. These reads were subsequently assembled using Trinity (2.8.3) software to generate unigenes. The longest transcript of each unigene served as the representative, which was annotated using databases such as NCBI non-redundant protein sequences (NR), Swiss-Prot, the Kyoto Encyclopedia of Genes and Genomes (KEGG), and clusters of orthologous groups for eukaryotic complete genomes (KOG). Gene expression levels were estimated using reads per kilobase per million (RPKM). Differential gene expression was analyzed using EdgeR (3.16.5) software; genes were identified as differentially expressed (DEGs) if they showed a log2(Fold Change) greater than 1 or less than -1 and a false discovery rate (FDR) below 0.05. Then, gene ontology (GO) analysis and KEGG enrichment analysis were performed for these DEGs. The homologous sequences of the target gene were retrieved from the NCBI database (https://www.ncbi.nlm.nih.gov) and aligned using MEGA11 software. A phylogenetic tree of the target gene was then constructed using the maximum likelihood method in MEGA11. All visualizations were created using GraphPad Prism (9.5.1) software and R (3.3.2) software.

### 2.7. qRT-PCR Analysis

The 10 μL of extracted RNA from each sample was absorbed with a pipette gun, and 2 μL of Oligo(-dT) (Takara, Kyoto, Japan) was added. The solution was then centrifuged and mixed, reacted at 70 °C for 10 min, and subsequently frozen for 2 min. Subsequently, 4 μL of 5× Reverse Transcriptase M-MLV Buffer (Takara, Japan) was added to the solution obtained in the preceding step. A total of 1 μL of Recombinant RNase inhibitor (Takara, Japan), 2 μL of dNTP (Takara, Japan) and 1 μL of Reverse Transcriptase M-MLV (RNase H-) (Takara, Japan) were reacted at 42 °C for 60 min and held at 70 °C for 15 min. The cDNA obtained was stored at −20 °C. A real-time fluorescence quantitative polymerase chain reaction (qRT-PCR) was conducted using TB Green^®^ Premix Ex Taq™ II Fast qPCR (Takara, Japan). The reaction system and procedure were set in accordance with the instructions provided, with the annealing temperature set at 60 °C. The 18S gene was selected as an internal control for normalizing the expression of the genes detected, as its expression levels were found to be more stable than those of the transcripts among the samples [6]. The 2^−ΔΔCt^ method was used to calculate the level of gene expression in each group. The specific primer pairs used are listed in Table S2. The data were analyzed and plotted using GraphPad Prism (9.5.1.)

## 3. Results 

### 3.1. The Relative Contents of Three Rotenoids Under Drought Stress

In this study, UPLC–ESI–MS/MS technology was employed to identify rotenoids in the drought-treated samples; the retention times for boeravinone E, boeravinone B, and boeravinone A were 6.61 min, 8.23 min, and 10.02 min, respectively (Figure 1B). The relative contents of the three rotenoids decreased first and subsequently increased as the drought severity increased due to the extension of time, but they remained below the initial levels observed in the control group (Figure 1C). Compared with the CK group, the relative contents of boeravinon E, boeravinon B, and boeravinon A in group M decreased by 93.00%, 85.14%, and 93.44%, respectively, after 5% PEG6000-simulated drought induction for 20 h (Figure 1C). After PEG6000-simulated drought induction for 40 h, the relative contents of boeravinon E, boeravinon B, and boeravinon A in group H recovered to 94.58%, 53.56%, and 37.78%, respectively (Figure 1C). Notably, boeravinon E consistently exhibited higher relative content than the other two rotenoids regardless of environmental conditions (Figure 1C). In the CK, the relative content of boeravinon E was 2.7-fold higher than that of boeravinon B and 3.0-fold higher than that of boeravinon A (Figure 1C). In group M, the relative content of boeravinon E was 1.3-fold higher than that of boeravinon B and 3.2-fold higher than that of boeravinon A. In group H, the relative content of boeravinon E was 4.8-fold higher than that of boeravinon B and 8.4-fold higher than that of boeravinon A. Furthermore, when subjected to mild drought stress, there was a significant decrease in the relative content of boeravinon E compared to the control group (Figure 1C).

### 3.2. Transcriptome Sequencing Assembly, Annotation, and Enrichment Analysis

**Figure 2:**
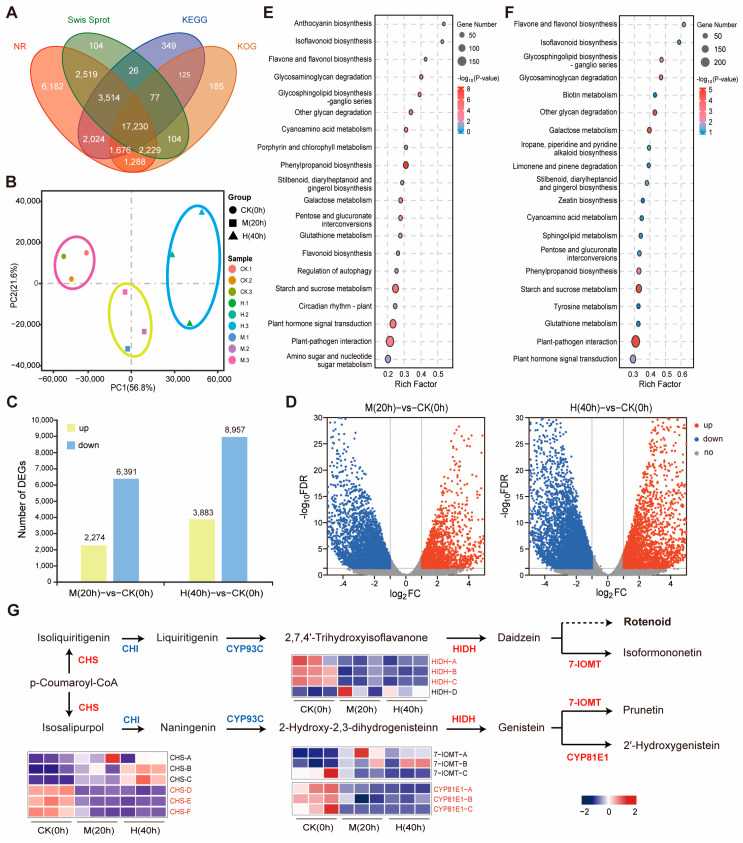
Gene functional annotation, analysis of the relationship between samples, and KEGG enrichment analysis of DEGs. (**A**) Venn plot of the genes annotated in the NR, Swiss Prot, KEGG, and KOG databases; (**B**) PCA between samples; (**C**) the number of differentially expressed genes (DEGs) in different comparison groups; (**D**) volcano maps of DEGs of M vs. CK and H vs. CK; (**E**) M vs. CK DEGs KEGG enrichment bubble map; (**F**) H vs. CK DEGs KEGG enrichment bubble map; (**G**) Analysis of the isoflavonoid biosynthesis pathway in *M. himalaica* associated with the three treatment groups under drought stress. The solid arrow indicates relationships between molecules in the biosynthetic pathway and activation in the signaling pathway, and the dashed arrow indicates indirect connections. Gene expression levels are presented based on the mean RPKM value from three biological replicates, which were log2 transformed and normalized. The color scale from blue to red represents the expression level of DEGs from low to high. CHS: chalcone synthase; CHI: chalcone isomerase; CYP93C: 2-hydroxyisoflavanone synthase; HIDH: 2-hydroxyisoflavanone dehydratase; 7-IOMT: isoflavone-7-O-methyltransferase; CYP81E: isoflavone 2′-hydroxylase.

All transcriptome analyses of the three treatments, CK, M, and H, yielded a total of 425,566,850 bp clean reads from nine samples, with Q20 > 97.80% and Q30 > 93.57% (Table S3), indicating high base recognition and good sequencing quality. Trinity splicing generated 119,404 unigenes with maximum, minimum, average, and N50 lengths of 16,836 bp, 201 bp, 835 bp, and 1397 bp, respectively. The mean guanine–cytosine (GC) content was 37.08% (Table S4). Numbers of unigenes successfully annotated using the NR, Swiss Prot, KEGG, and KOG databases were 36,662 (30.70%), 25,803 (21.61%), 25,021 (20.95%), and 22,914 (19.19%), respectively. A total of 37,632 genes were annotated in at least one of these databases (Figure 2A and Figure S1).

Correlation heatmap analysis showed that the Pearson correlation coefficient (PCC) was between 0.521~1.000, and PCC among the biological replicates in this experiment was >0.890 (Figure S2). Principal component analysis (PCA) results showed that the first principal component (PC1) and the second principal component (PC2) accounted for 56.8% and 21.6% of the variation, respectively, and the samples were clustered clearly (Figure 2B). The above results showed that the intra-group repeatability and inter-group specificity were good.

In order to elucidate the response of *M. himalaica* to drought stress, a negative binomial generalized log-linear model was employed as the criterion for screening differentially expressed genes (DEGs) in response to CK, M, and H treatments, with FC > 1 and FDR < 0.05. A total of 14,525 DEGs were identified in the comparison group of M_vs_CK and H_vs_CK, consisting of 4505 upregulated and 10,020 downregulated genes (Figure S1). Among these DEGs, 6980 genes (1652 upregulated and 5328 downregulated) were commonly expressed across all groups (Figure 2C,D).

The DEGs between the M and H treatment groups and the control group were statistically analyzed by GO annotation. In both M_vs_CK and H_vs_CK, genes associated with binding and catalytic activity in the category “molecular function” were the most abundant (Tables S5 and S6). KEGG pathway enrichment analysis was subsequently performed on the entire set of DEGs. Under drought stresses, some common pathways were enriched in both M and H groups, such as “plant-pathogen interaction”, “plant hormone signal transduction”, “starch and sucrose metabolism”, “phenylpropanoid biosynthesis”, “flavone and flavonol biosynthesis”, and “isoflavonoid biosynthesis” (Figure 2E,F).

### 3.3. Analysis of Isoflavonoid Biosynthesis Pathway Involved in Drought Stress Responses

Rotenoids, belonging to the isoflavone family, serve as the principal active constituents of *M. himalaica*. To elucidate the response of isoflavone biosynthesis to drought stress, extensive screening and pathway mapping of the DEGs potentially involved in this process were conducted (Figure 2G). In the isoflavone biosynthesis pathway, p-Coumaroyl-CoA serves as a metabolic substrate that generates isoflavones via two distinct branches. The naringenin branch leads to the production of isoflavones such as prunetin and 2′-hydroxygenistein. The liquiritigenin branch produces malonyldaidzin, isoformononetin, and rotenoid compounds. The gene family with the largest number (four genes) of enriched genes was 2-hydroxyisoflavanone dehydratase (HIDH), which exhibited different expression patterns under two drought conditions. Homologous sequences of the four HIDH genes were then identified and used to construct a phylogenetic tree (Figure S3) [16]. Among the isoflavonoid biosynthesis genes, three *HIDH* genes (*HIDH-A*, *HIDH-B,* and *HIDH-C*), three isoflavone 2′-hydroxylase genes (*CYP81E1-A*, *CYP81E1-B,* and *CYP81E1-C*), one isoflavone-7-O-methyltransferase gene (*7-IOMT-C*), and one isoflavone 7-O-glucoside-6″-O-malonyltransferase gene (*IF7MAT*) were downregulated under drought stress, while one *HIDH* gene (*HIDH-D*) and two *7-IOMT* gnens (*7-IOMT-A* and *7-IOMT-B*) were upregulated. Moreover, the *7-IOMT-B* gene had the highest expression under the two drought stress treatments, compared with other genes.

### 3.4. Analysis of ABA, Auxin, and JA Biosynthesis During the Drought Stress Responses

**Figure 3:**
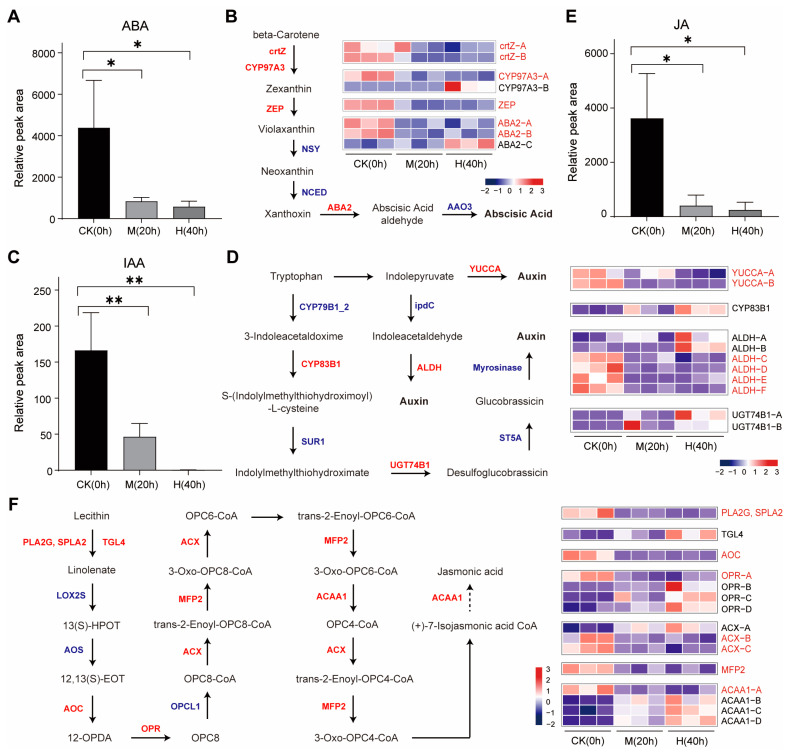
Analysis of the ABA, auxin, and JA biosynthesis pathway in *M. himalaica* under drought stress. (**A**) The relative content of ABA; (**B**) analysis of ABA biosynthesis pathway in *M. himalaica* associated with the three treatment groups under drought stress; (**C**) the relative content of IAA; (**D**) analysis of auxin biosynthesis pathway in *M. himalaica* associated with the three treatment groups under drought stress; (**E**) the relative content of JA; (**F**) analysis of the JA biosynthesis pathway in *M. himalaica* associated with the three treatment groups under drought stress. The solid arrow indicates relationships between molecules in the biosynthetic pathway and activation in the signaling pathway, and the dashed arrow indicates indirect connections. Gene expression levels are presented based on the mean RPKM value from three biological replicates, which were log2 transformed and normalized. The color scale from blue to red represents the expression level of DEGs from low to high. crtZ: β-carotene 3-hydroxylase; CYP97A3: β-ring hydroxylase; ZEP: zeaxanthin epoxidase; NSY: neoxanthin synthase; NCED: 9-cis-epoxycarotenoid dioxygenase; ABA2: xanthoxin dehydrogenase; AAO3: abscisic-aldehyde oxidase; YUCCA: indole-3-pyruvate monooxygenase; ipdC: indolepyruvate decarboxylase; ALDH: aldehyde dehydrogenase; CYP79B1_2: tryptophan N-monooxygenase; CYP83B1: aromatic aldoxime N-monooxygenase; SUR1: S-alkyl-thiohydroximate lyase SUR1; UGT74B1: N-hydroxythioamide S-β-glucosyltransferase; ST5A: aromatic desulfoglucosinolate sulfotransferase; PLA2G, SPLA2: secretory phospholipase A2; PLA2G16: HRAS-like suppressor 3; LOX2S: lipoxygenase; AOS: hydroperoxide dehydratase; AOC: allene oxide cyclase; OPR: 12-oxophytodienoic acid reductase; ACX: acyl-CoA oxidase; MFP2: enoyl-CoA hydratase/3-hydroxyacyl-CoA dehydrogenase; ACCA1: acetyl-CoA acyltransferase. Statistical analysis was performed using one-way ANOVA. Asterisks * and ** indicate statistical significance at *p* < 0.05 and *p* < 0.01, respectively.

Plant hormone signal transduction pathways were enriched in *M. himalaica* calli after drought treatment. Previous studies have revealed that abscisic acid (ABA), auxin, and JA have been identified as critical mediators of drought resistance processes [11,12,13,14,17]. Thus, the biosynthesis of ABA, auxin, and JA were investigated in drought-stress-treated calli of *M. himalaica*. The endogenous ABA levels in M. himalaica calli were markedly decreased following exposure to drought stress (Figure 3A). The ABA biosynthesis pathway begins with β-carotene and progresses through several important intermediates [18], including xanthoxin and ABA aldehyde, before culminating in the synthesis of ABA (Figure 3B). We identified a total of eight genes across four gene families; of these, six genes were downregulated and the other two, encoding the β-ring hydroxylase (*CYP97A3-B*) and xanthoxin dehydrogenase (*ABA2-C*), were upregulated under drought stress (Figure 3B). Also, the levels of endogenous auxin and JA in *M. himalaica* calli were significantly reduced following drought stress exposure (Figure 3C,E). The pathway for auxin biosynthesis initiates with tryptophan, and, under drought stress, the expression of encoding two indole-3-pyruvate monooxygenase (*YUCCA)* genes and aldehyde dehydrogenase (*ALDH*) genes was notably suppressed (Figure 3D). Similarly, the JA biosynthetic pathway originates from lecithin, and the expression levels of one gene, one allene oxide cyclase (*AOC*) gene, one 12-oxo-phytodienoic acid reductase (*OPR*) gene, two acyl-CoA oxidase (*ACX*) genes, one multifunctional protein gene (*MFP2*), and one acetyl-CoA acyltransferase 1 (*ACAA1*) gene were significantly downregulated during drought stress (Figure 3F).

### 3.5. Analysis of the ABA, Auxin, and JA Signal Transduction Pathway Involved in Drought Stress Responses

**Figure 4:**
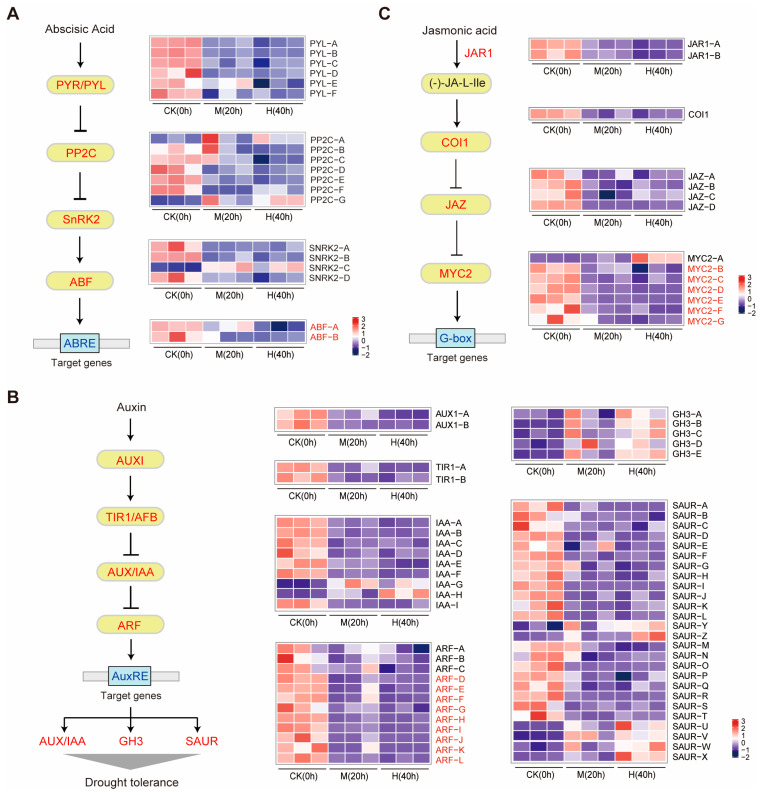
Analysis of the ABA, auxin, and JA signal transduction pathway in M. himalaica under drought stress. (**A**) Analysis of the ABA signal transduction pathway in *M. himalaica* associated with the three treatment groups under drought stress; (**B**) analysis of the auxin signal transduction pathway in *M. himalaica* associated with the three treatment groups under drought stress; (**C**) analysis of the JA signal transduction pathway in *M. himalaica* associated with the three treatment groups under drought stress. The solid arrow indicates activation in the signaling pathway, and the vertical solid line with a short transverse solid line at the end indicates inhibition. Gene expression levels are presented based on the mean FPKM value from three biological replicates, which were log2 transformed and normalized. The color scale from blue to red represents the expression level of DEGs from low to high. PYR/PYL: pyrabactin resistance 1/pyrabactin-resistance-like; PP2C: protein phosphatase 2C; SNRK2: sucrose nonfermenting related kinases 2; ABF: ABA response element binding factor; ABRE: abscisic acid response element; AUX1: auxin influx carrier; TIR1: transport inhibitor response 1; IAA: auxin-responsive protein IAA; ARF: auxin response factor; CH3: auxin responsive GH3 gene family; SAUR: SAUR family protein; JAR1: jasmonic acid-amino synthetase; COI-1: coronatine-insensitive protein 1; JAZ: jasmonate ZIM domain-containing protein; MYC2: transcription factor MYC2.

To further explore the mechanism of drought stress in the calli of *M. himalaica*, the gene expression profiles in the signaling pathway of the ABA, auxin, and JA signal transduction pathways were investigated. The ABA receptor pyrabactin resistance 1/pyrabactin resistance-like (PYR/PYL), protein phosphatase 2C (PP2C), sucrose nonfermenting related kinases 2 (SNRK2), and ABA response element binding factor (ABF) are now known to be the core element of ABA perception and signal transduction [11,19] (Figure 4A). In the drought-treated *M. himalaica*, we identified a total of 19 genes belonging to four gene families, 16 of which were downregulated under drought stress (Figure 4A). The gene family enriched *PP2C* exhibited different expression patterns under two drought conditions. For the seven *PP2C* genes, five were markedly downregulated under drought stress, while two genes (*PP2C-A* and *PP2C-G*) were highly upregulated (Figure 4A). In addition, four *SNRK2* genes, including three (*SNRK2-A*, *SNRK2-B,* and *SNRK2-D*) downregulated and one (*SNRK2-C*) upregulated, were found (Figure 4A). Finally, we also identified two *ABF* genes (*ABF-A* and *ABF-B*) that were downregulated under drought stress in *M. himalaica* (Figure 4A), which has the potential to regulate the rotenoid biosynthesis. The auxin signal transduction pathway encompasses key components such as auxin input carrier AUXIN1 (AUX1), auxin receptor TIR1/AFB, and early auxin response protein AUX/IAA, which can specifically bind to auxin response factor (ARF) (Figure 4B). ARFs can interact with the AuxRE binding element on target genes, including *AUX/IAA*, *GH3*, and *SAUR* genes, which are known to enhance drought tolerance [20]. Under drought stress conditions in *M. himalaica* calli, the expression levels of two *AUX1* genes, two *TIR1* genes, seven *IAA* genes, and nine *ARF* genes were significantly downregulated, whereas all five *GH3* genes exhibited upregulation in response to severe drought stress (Figure 4B). The JA signal transduction pathway encompasses key components such as JAR1, which could transform the JA to its active form (-)-JA-L-Ile, coronatine-insensitive 1 (COI1), jasmonate ZIM-domain proteins (JAZ), and MYC2, which can specifically bind to the target genes containing the G-box binding element (Figure 4C). The expression levels of two *JAR1* genes, one *COI1* gene, four *JAZ* genes, and six *MYC2* genes were notably suppressed, indicating significant downregulation in response to the drought stress in *M. himalaica* calli (Figure 4C).

### 3.6. Quantitative Real-Time RT-PCR Analysis

**Figure 5:**
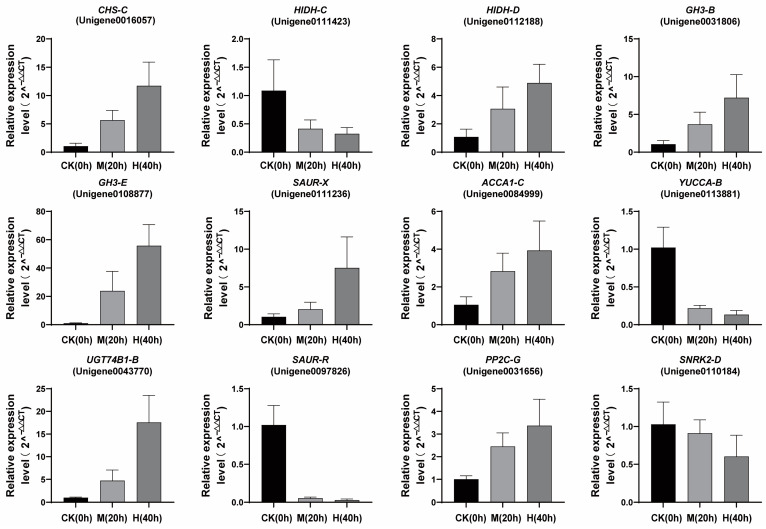
Quantitative analysis of genes related to *M. himalaica* responses to drought stress.

To ascertain the veracity of the transcriptome results and ascertain the expression patterns of the aforementioned differentially expressed genes (DEGs) under varying degrees of drought stress, 12 randomly selected DEGS were subjected to a quantitative reverse transcription polymerase chain reaction (qRT-PCR) (Figure 5). The results demonstrated that the expression of genes *CHS-C* (Unigene0016057) and *HIDH-D* (Unigene0112188), which are involved in the biosynthesis of rotenoids, was upregulated, whereas the expression of *HIDH-C* (Unigene0111423) was downregulated. This was consistent with the findings of the transcriptome sequencing performed. In the ABA signaling pathway, the gene *PP2C-G* (Unigene0112133) exhibited increased expression, while the gene *SNRK2-D* (Unigene0110184) displayed decreased expression. Similarly, in the IAA biosynthesis pathway, the expression of the *UGT74B1-B* gene (Unigene0043770) was increased, while that of the *YUCCA-B* gene (Unigene0113881) was decreased. It is noteworthy that, in the IAA signaling pathway, all of the genes quantified by qRT-PCR exhibited increased expression, with the exception of the *SAUR-R* gene (Unigene0097826). The results demonstrated that the expression of *ACAA1-C* (Unigene0084999), which is involved in the biosynthesis of jasmonic acid (JA), was upregulated. In general, the results of qPCR analysis coincided with the findings of transcriptome sequencing, thereby demonstrating the reliability of transcriptome sequencing.

## 4. Discussion

*M. himalaica*, mainly distributed in arid areas with little rain, is a traditional herb in Xizang, China. Rotenoids, as isoflavone compounds, are the main medicinal active components of *M. himalaica*, known for their significant anticancer effects. Advances in multi-omics approaches have improved our understanding of plant resistance to abiotic stresses, particularly drought stress. In this study, 5% PEG6000 was used to treat calli of *M. himalaica* to simulate drought stress, as previously reported in wheat [21], *Passiflora edulis* Sims [22], and Tartary buckwheat (*Fagopyrum tararicum*) [23]. Subsequently, the relative contents of the three rotenoids were determined, and transcriptome technology was used to explore the effects of drought stress on biosynthesis of rotenoids, as well as the coping strategies and reaction mechanisms of *M. himalaica* under drought stress.

Our findings showed that the relative contents of all three rotenoids in the calli of *M. himalaica* were reduced under drought treatment, and the expression of genes related to their synthesis was also significantly reduced. However, a previous study showed that drought stress led to a significant increase in flavonoids, such as matricin, nehesperidin, and hypericin, in drought-tolerant sour orange leaves, while these compounds decreased or showed no significant change in drought-sensitive lemon leaves [24]. Furthermore, research has demonstrated that treatment with 5% and 10% PEG 6000 significantly increased the content of four types of flavonoids in *Scutellaria baicalensis*, whereas treatment with 15%, 20%, and 25% PEG 6000 significantly decreased the content of these flavonoids [25]. It has also been observed that prolonged drought during various stages of soybean seed development significantly reduces the isoflavone content in seeds [26]. We hypothesize that rotenoid levels declined due to three primary reasons. First, callus tissue served as the plant material in this experiment. Unlike plants in natural settings, the calli of *M. himalaica* are grown in a controlled and simplified environment, which might result in distinct secondary metabolite profiles. Secondly, under drought conditions, plants typically lower their overall metabolic rate to conserve water and energy [27], which might result in decreased synthesis of rotenoids. Finally, rotenoids are only one of the isoflavonoid components, and the decrease in rotenone content may be related to the increased expression of genes upstream of its synthesis pathway that are involved in the biosynthesis of isoflavones or flavonoids (Figure 3A).

Plant hormones are important mediators of plant response to environmental stresses, including drought stress. Drought stress induces a series of complex plant hormone regulatory networks, including ABA [17,28], auxin [29], JA [14], ethylene [30], gibberellin [31], etc. Among these, ABA, auxin, and JA are predominant hormones in the response to drought stress [19]. This may be related to significantly increased ABA levels in plants under drought conditions, resulting in reduced stomatal closure and water loss [28], as well as the regulation of gene expression associated with enhanced drought tolerance [19,32]. Additionally, auxin and JA could participate in the positive regulation of drought stress resistance by the activation of some drought-responsive genes and some secondary plant metabolic pathways [33,34]. The plant hormone signal transduction pathway was significantly enriched under different drought treatments, and 8 and 19 DEGs related to ABA biosynthesis and signaling pathways were identified, respectively. Under drought stress, most DEGs related to ABA synthesis and ABA signaling were downregulated in *M. himalaica* treated calli. In the ABA biosynthesis pathway, we identified eight DEGs in four gene families. Among them, two genes (*CYP97A3* and *ABA2-C*) were upregulated under drought stress, and the other six genes were downregulated under drought stress (Figure 3B). Additionally, it has been shown that overexpression of *AtNCED3* in *Arabidopsis* causes an increase in endogenous ABA levels and promotes transcription of drought and ABA-induced genes, playing a key role in ABA biosynthesis [35]. Moreover, the increase in ABA level in Arabidopsis seeds under high-temperature conditions was associated with the up-regulation of zeaxanthinine cyclooxygenase gene *ABA1/ZEP* and three 9-cis-epoxide carotenoid dioxygenase genes *NCED2*, *NCED5*, and *NCED9* [36]. In our research on the effects of drought stress on *M. himalaica*, we observed significant inhibition of the expression of two *YUCCA* genes and an *ALDH* gene involved in the auxin biosynthesis pathway (Figure 3D). Furthermore, it was observed that the expression of the majority of genes within the auxin signal transduction pathway was markedly reduced, while the expression of five *GH3* genes was significantly increased (Figure 4B). Similarly, most genes involved in JA biosynthesis and signal transduction pathways were downregulated, such as *ACX* genes, *JAR1* genes, *JAZ* genes, and *MYC2* genes (Figure 3F and Figure 4C). In summary, we hypothesized that downregulation of the expression of genes regulating ABA, auxin, and JA biosynthesis would lead to a decrease in their endogenous content in the plant body and consequently affect ABA signaling.

**Figure 6:**
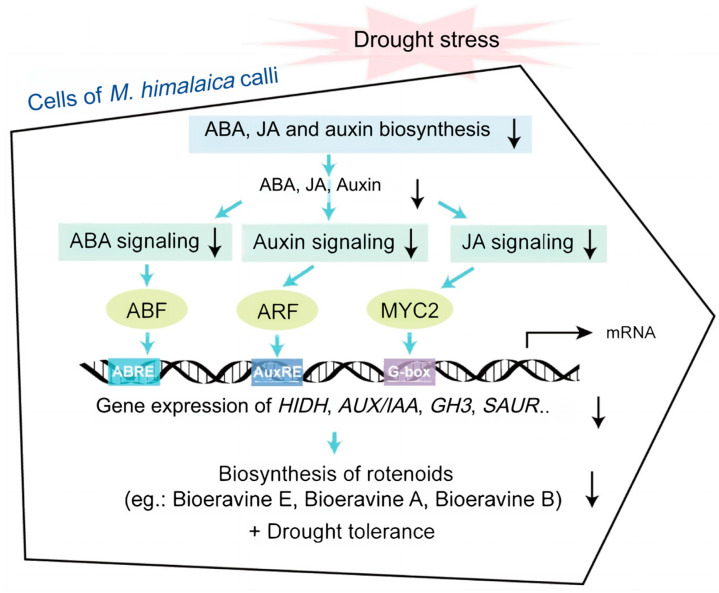
Theoretical molecular mechanism in *M. himalaica* in response to drought stress. ABA: abscisic acid; ARF: auxin response factor; AuxRE: auxin response element; HIDH: 2-hydroxyisoflavanone dehydratase. The black arrows indicate a drop in content or expression, the blue arrows indicate the flow (next level reaction), and the black broken line arrows indicate transcription and translation.

In the ABA signaling transduction pathway, all six *PYR/PYL* genes (*PYL-A*, *PYL-B*, *PYL-C*, *PYL-D*, *PYL-E*, and *PYL-F*) were continuously downregulated under drought stress, while five *PP2C* genes were markedly downregulated, and two genes (*PP2C-A* and *PP2C-G*) were highly upregulated. Additionally, four *SnRK2* genes were identified, with three (*SnRK2-A*, *SnRK2-B*, and *SnRK2-D*) downregulated and one (*SnRK2-C*) upregulated under drought stress. Furthermore, two *ABF* genes (*ABF-A* and *ABF-B*) were also downregulated (Figure 3C). Previous research found that under drought stress, ABA accumulation promoted the interaction between basic helix–loop–helix (bHLH) transcription factors and the ABA receptor PYL8. This interaction forms a complex that interferes with the repression of ABA-responsive genes by bHLH118, thereby activating the ABA response and enhancing drought tolerance [37]. The expression of *ZmPP2C-As* was significantly upregulated in response to drought in maize, suggesting *ZmPP2C-As* may play a crucial role in mediating the response to drought environmental stress [38]. At the same time, it has been reported that the emergence of PP2C in land plants is to inhibit the drought tolerance mechanism, and ABA releases the inherent drought tolerance in plants by regulating PP2C [39]. In drought-tolerant mulberry trees, components of the ABA signaling pathway, including three *PYLs*, two *PP2Cs*, two *SnRK2s*, four *ABFs*, and the ABA-responsive gene *MaRD29B*, were identified, with the γ subunit of the mulberry heterotrimeric G protein regulating the pathway in response to drought by interacting with PP2Cs and SnRK2s [40]. Overexpression of *AtABF3*, a bZIP transcription factor named ABSCISIC ACID-RESPONSIVE ELEMENT-BINDING FACTOR 3 (ABF3), significantly enhanced the tolerance of transgenic alfalfa to drought stress manifested by lower cell membrane damage and higher chlorophyll content, along with a reduction in leaf size, which may help to reduce water loss and improve drought tolerance in plants [41]. ARFs are a class of transcription factors that recognize and bind to auxin response element (AuxRE) to regulate the expression of response genes [42]. ARFs regulate the expression of genes determining the state of their structural existence, which can form dimers with other ARFs or growth hormone deterrent proteins Aux/IAA to block the transcriptional activity of ARF [43]. At the same time, numerous studies have demonstrated that *HIDH* is a key gene regulating flavonoid biosynthesis [44,45,46]. MYC2, a fundamental regulator of the JA signaling pathway, is also engaged in drought resistance and the biosynthesis of secondary metabolites involved in terpenoids, alkaloids, flavonoids, and thioglycosides [47]. Additionally, research has shown that when licorice seedlings face drought stress, both *HIDH* gene expression and protein levels increase, along with an elevation in flavonoid metabolism and the content of superoxide scavenging enzymes [48]. In summary, we hypothesized that the decrease in ABA, auxin, and JA content and their weakened signaling under drought stress regulated the reduced expression of rotenone biosynthesis-related genes, such as *HIDH*, through ABFs, ARFs, and MYC2, which led to a decrease in their expression and thus reduced the content of the three rotenoids, boeravinone E, boeravinone B, and boeravinone A (Figure 6).

In this study, we used transcriptome analysis for the first time to elucidate the drought resistance mechanism of *M. himalaica* calli in a PEG6000-simulated drought environment. The transcriptomic analysis revealed that 14,525 DEGs were responsive to drought stress. Through functional annotation and KEGG enrichment analysis of the DEGs, we identified key pathways associated with drought stress, including plant–pathogen interactions, plant hormone signaling, starch and sucrose metabolism, and isoflavone biosynthesis. Furthermore, this study mapped out the DEGs involved in isoflavone biosynthesis while also identifying the key genes in isoflavone biosynthesis, such as *CYP81E1*, *IF7MAT*, *HIDH* and *7-IOMT*, responsible for responses to drought stress. Our study reveals that drought stress significantly reduces the content of rotenoids in *M. himalaica* and alters the hormonal balance, particularly affecting ABA, auxin, and JA signaling. These changes not only influence drought tolerance but also the accumulation of secondary metabolites. We subsequently elucidated the ABA, auxin, and JA synthesis and signaling pathways that are potentially implicated in the regulation of flavonoid biosynthesis. Overall, the findings of this study provide valuable insights into the molecular mechanisms by which *M. himalaica* responses to drought (Figure 6). This work can be used to develop targeted breeding strategies to develop drought-resistant *M. himalaica* varieties. The screening and analysis of genes (e.g., CHS, HIDH, GH3, etc.) involved in the response to drought stress and the accumulation of rotenone can facilitate the elucidation of the molecular mechanisms underlying the behavior of plants under conditions of drought. The screening and analysis of genes (e.g., *CHS*, *HIDH*, *GH3*, etc.) involved in the response to drought stress and the accumulation of rotenone can facilitate the elucidation of the molecular mechanisms underlying the behavior of plants under conditions of drought. The introduction of these genes into the breeding process, for example, through the use of molecular-marker-assisted selection (MAS) methods, allows for the rapid screening of individuals exhibiting the desired traits. By employing MAS, breeders can select varieties that exhibit excellent drought tolerance and high metabolite accumulation through genotypic screening at an early stage, obviating the need to await field performance at a subsequent stage. The breeding of varieties capable of performing better in drought environments can be accelerated. This will not only enhance the crop’s drought tolerance but also mitigate yield losses attributable to water deficit. Based on these screened key genes, breeders can precisely regulate their expression using gene-editing techniques (e.g., CRISPR-Cas9) or transgenic technologies. This not only accelerates the breeding of drought-resistant varieties but also improves breeding efficiency by avoiding the gene dragging effect that may occur in traditional breeding methods. Future studies should validate the hypotheses using metabolomics to assess key metabolite levels (e.g., isoflavones) and their correlation with gene expression, as well as proteomics to examine protein abundance and modifications. These complementary techniques will enhance our understanding of how drought stress affects *M. himalaica* at multiple molecular levels. While this study provides valuable insights into the drought resistance mechanisms of *M. himalaica*, one limitation is that it relies on in vitro calli, which may not fully represent the plant’s response under natural environmental conditions. Future research should address this gap by conducting field trials to validate the findings in whole plants.

## 5. Conclusions

This study utilized transcriptome analysis to elucidate the drought resistance mechanisms of *M. himalaica* calli under PEG6000-simulated drought conditions for the first time. We identified 14,525 DEGs responsive to drought stress, with functional annotation and KEGG enrichment revealing key pathways such as plant–pathogen interactions, hormone signaling, starch and sucrose metabolism, and isoflavone biosynthesis. Key genes like *CYP81E1*, *IF7MAT*, *HIDH*, and *7-IOMT* were highlighted in the isoflavone biosynthesis pathway. Additionally, ABA, auxin and JA synthesis and signaling pathways were potentially implicated in isoflavone biosynthesis. These findings provide valuable insights for developing drought-resistant *M. himalaica* varieties through targeted breeding strategies.

## Data Availability

Data are contained within the article and Supplementary Materials.

## Supplementary Materials

The following supporting information can be downloaded at https://www.mdpi.com/article/10.3390/genes15121644/s1, Figure S1: The number of unigenes successfully annotated in the four databases; Figure S2: Correlation heat map between samples; Figure S3: Gene evolution tree of *HIDH* gene and its homologous genes; Table S1: Three endogenous plant hormone information; Table S2: qRT-PCR primers used to validate the genes and internal references; Table S3: Illumina sequencing data pre-processing statistics; Table S4: Assembly quality statistics; Table S5: Gene Ontology enrichment analysis for differentially expressed genes in M compared to CK; Table S6: Gene Ontology enrichment analysis for differentially expressed genes in H compared to CK.
