# Supplementary material for: Drought Stress Inhibits the Accumulation of Rotenoids and the Biosynthesis of Drought-Responsive Phytohormones in Mirabilis himalaica (Edgew.) Heim Calli
Source: Genes (Basel). 2024 Dec 21;15(12):1644. doi: 10.3390/genes15121644 (PMC11675678; doi:10.3390/genes15121644)
Supplement: Supplementary file 1 [file genes-15-01644-s001.zip › genes-3328088-supplementary.pdf]

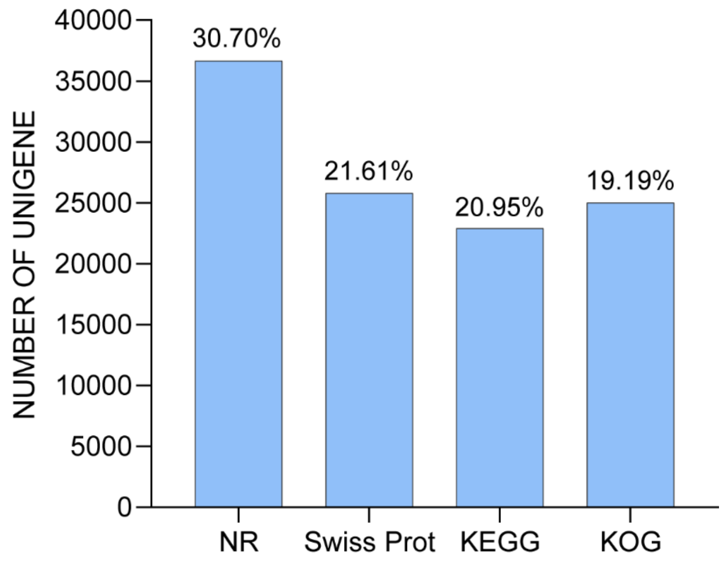

Figure S1. Transcriptome sequencing annotation by the NR, Swiss Prot, KEGG, and KOG databases.

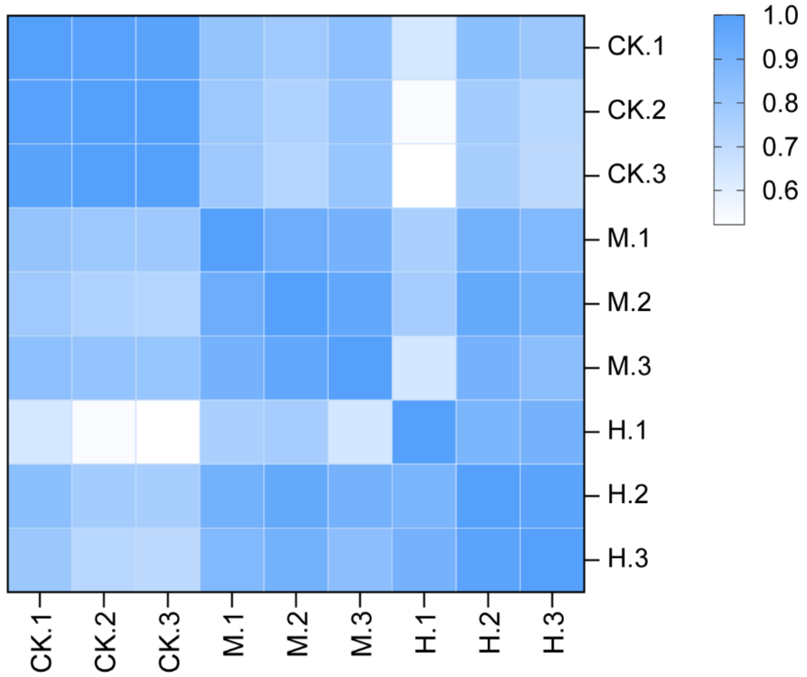

Figure S2. Pearson correlation analysis among the biological replicates in this study.

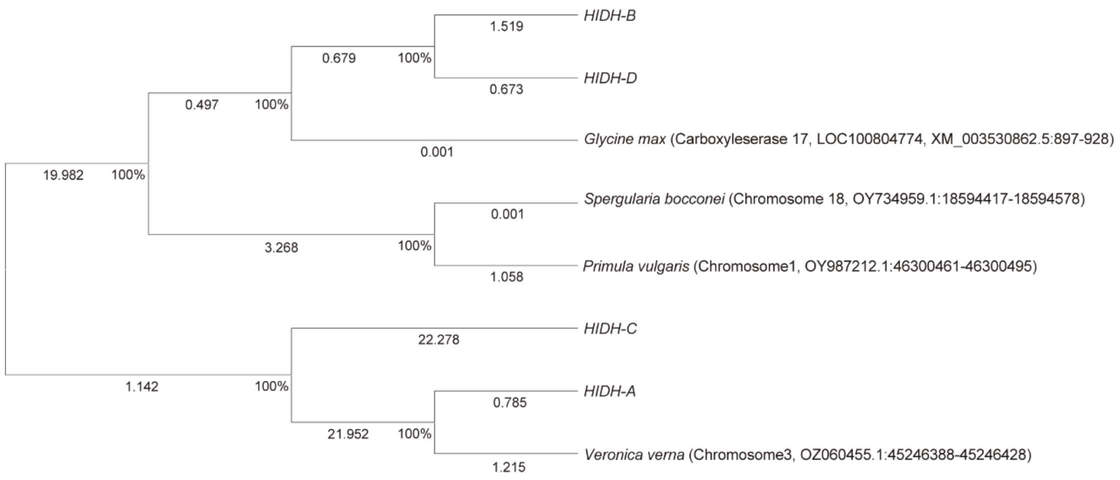

Figure S3. The evolution tree of *HIDH* genes constructed by the maximum likelihood method in MEGA software.

Table S1. Three endogenous plant hormone information.

Compound	Molecular formula	Relative molecular mass	Chemical Abstracts Service Number	Retention time(min)
Abscisic acid	$C_{15}H_{20}O_4$	264.1362	14375-45-2	1.82
Auxin	$C_{10}H_9NO_2$	175.0633	87-51-4	1.13
Jasmonic acid	$C_{12}H_{18}O_3$	210.1256	77026-92-7	0.85

Table S2. qRT-PCR primers used to validate the genes and internal references.

NO.	Unigene Id	Gene	Forward	Reverse
1	Unigene0027948	*CHS-C*	CCGTGACATTGCCGCACTTCT	GACCAGCATGGCAGCCTACAAG
2	Unigene0111423	*HIDH-C*	GCAGGAGGAAGCATCACGCATC	TAGCCACCTCATCGCCAATCCG
3	Unigene0112188	*HIDH-D*	TGTCTCCGCCAGTTCCAGCAA	CGAGTTTGGGCAGCCAAATTGT
4	Unigene0031806	*GH3-B*	TGGCCCAACAAAGGAGGTTT	CAGTGAGGGAAACATGGGCT
5	Unigene0108877	*GH3-E*	AGTTCCAAGATGTGTTAGTGCT	CAAAGATGGGCTGAAATGGGC
6	Unigene0111236	*SAUR-X*	GCACTTGCCTCATCTTCTCCATCA	GTTCCTCTCCTTCATGTCCTACCA
7	Unigene0084999	*ACCA1-C*	CCTGTACCGACGAAGGTTGTTGA	GCTCCAGCACCATCAGTCACTT
8	Unigene0113881	*YUCCA-B*	CGGCAATAACAAGCGTAAGAGGAA	GCATCCATAGCGGCTCCATAGA
9	Unigene0043770	*UGT7B1-B*	CGGGCTGATTGGGTGTTTTG	CCCATGCCAGTTCCTCCATT
10	Unigene0097826	*SAUR-R*	TGATGAACCACGAGCCCAAG	ACCACCATTCTCTCAGCAGC
11	Unigene0112133	*PP2C-G*	GCTCCGTCAACTGCGAGTCATC	CCGTCGTTACCATTCCGTTCGT
12	Unigene0110184	*SNRK2-D*	TGCCAAGGGAACTCACCGAACA	TGACACCGGAGGTGGCTTCTT
13	18s		ATGATAACTCGACGGATCGC	CTTGGATGTGGTAGCCGTTT

Table S3. Illumina sequencing data pre-processing statistics.

Sample	Raw reads	Clean reads	Percent (%)	Clean bases	Mapped reads (%)	Q20 (%)	Q30 (%)
CK.1	47348754	46939366	99.14	7024280403	98.90	97.94	93.85
CK.2	48508128	48143948	99.25	7198104390	98.93	98.15	94.38
CK.3	48403362	48032736	99.23	7190513141	99.04	98.19	94.44
M.1	48455736	48070012	99.20	7192285948	98.95	98.13	94.32
M.2	47789420	47448680	99.29	7100965214	99.06	98.2	94.50
M.3	46672966	46058992	98.68	6895673270	98.50	97.95	93.95
H.1	47395842	46801888	98.75	7006432902	98.55	97.8	93.57
H.2	47126352	46769054	99.24	7004231731	99.08	98.07	94.20
H.3	47661388	47302174	99.25	7087760616	99.14	98.03	94.09

Table S4. Assembly quality statistics.

Genes Number	GC (%)	Max length	Min length	Average length	N50	Total assembled bases
119405	37.085	16863	201	835	1397	99723727

Table S5. Gene Ontology enrichment analysis for differentially expressed genes in M compared to CK.

Ontology	Class	Number of up-regulated genes	Number of down-regulated genes
Biological Process	response to stimulus	44	191
Biological Process	signaling	5	43
Biological Process	single-organism process	102	322
Biological Process	developmental process	10	58
Biological Process	biological regulation	45	150
Biological Process	metabolic process	127	361
Biological Process	cellular component organization or biogenesis	17	84
Biological Process	localization	25	86
Biological Process	cellular process	129	405
Molecular Function	nucleic acid binding transcription factor activity	11	69
Molecular Function	catalytic activity	198	622
Molecular Function	transporter activity	11	105
Molecular Function	binding	206	673
Cellular Component	membrane	98	560
Cellular Component	membrane part	89	496
Cellular Component	organelle part	31	129
Cellular Component	cell	122	495
Cellular Component	cell part	116	488
Cellular Component	macromolecular complex	18	59
Cellular Component	organelle	83	328

Table S6. Gene Ontology enrichment analysis for differentially expressed genes in H compared to CK.

Ontology	Class	Number of up-regulated genes	Number of down-regulated genes
Biological Process	response to stimulus	66	233
Biological Process	single-organism process	159	464
Biological Process	developmental process	24	88
Biological Process	multicellular organismal process	22	81
Biological Process	metabolic process	192	503
Biological Process	cellular component organization or biogenesis	39	120
Biological Process	localization	45	126
Biological Process	biological regulation	58	213
Biological Process	cellular process	204	572
Molecular Function	nucleic acid binding transcription factor activity	21	72
Molecular Function	catalytic activity	302	825
Molecular Function	transporter activity	24	140
Molecular Function	binding	320	883
Cellular Component	membrane	168	757
Cellular Component	membrane part	143	672
Cellular Component	organelle part	58	215
Cellular Component	cell	212	699
Cellular Component	cell part	206	690
Cellular Component	macromolecular complex	146	491
Cellular Component	organelle	83	328
